# Perioperative management of DOAC in elective orthopedic surgery: a prospective cohort analysis

**DOI:** 10.1097/MS9.0000000000005677

**Published:** 2026-10-06

**Authors:** Michael Axenhus, Paula Kelly-Pettersson, Emma Warholm, Rickard E. Malmström, Margareta Holmström, Inga Fredriksson, Olof Sköldenberg, Håkan Wallén, Eli Westerlund

**Affiliations:** a Department of Clinical Sciences at Danderyd Hospital, Division of Orthopaedics, Karolinska Institutet, Stockholm, Sweden; b Department of Internal Medicine, Danderyd Hospital, Stockholm, Sweden; c Department of Clinical Sciences at Danderyd Hospital, Division of Internal Medicine, Karolinska Institutet, Stockholm, Sweden; d Department of Medicine Solna, Division of Clinical Pharmacology, Karolinska Institutet, Stockholm, Sweden; e Department of Health, Medicine and Caring Sciences, Linköping University, Linköping, Sweden; f Department of Anesthesia and Intensive Care, Danderyd Hospital, Stockholm, Sweden; g Department of Clinical Sciences at Danderyd Hospital, Division of Cardiovascular Medicine, Karolinska Institutet, Stockholm, Sweden

**Keywords:** anticoagulation, DOACs (direct oral anticoagulants), orthopedic surgery, perioperative management, plasma concentration

## Abstract

**Objective:**

Treatment with direct oral anticoagulants is usually interrupted prior to surgery to reduce bleeding risk. Current guidelines recommend standardized interruption protocols, but the actual preoperative plasma levels and their clinical implications in real-world surgical populations are not well characterized. The aim of this study was to assess preoperative DOAC plasma concentrations and postoperative bleeding and thromboembolic outcomes in patients undergoing elective orthopedic surgery.

**Methods:**

We conducted a prospective observational study at Danderyd Hospital, Stockholm, Sweden. Adults (≥ 18 years) treated with apixaban, rivaroxaban, or dabigatran for atrial fibrillation or venous thromboembolism and scheduled for high-bleeding-risk orthopedic procedures were included. DOAC interruption followed national guidelines. Plasma DOAC levels were measured preoperatively using LC-MS/MS. Clinical outcomes, including bleeding and thromboembolic events, were recorded up to 30 days postoperatively.

**Results:**

A total of 54 patients receiving ongoing DOAC therapy (mean age, 75.5 years; 54% male) were included, of whom 87% were on apixaban. Plasma DOAC concentrations had a mean of 6.7 ng/mL (range, <1.9 to 23.1 ng/mL), and 38% of patients had levels below the limit of detection (< 2.0 ng/mL). One patient had a concentration >30 ng/mL. Five patients (16%) experienced clinically relevant non-major bleeding; four of these had undetectable DOAC levels. No major bleeding, thromboembolic events, or deaths were observed.

**Conclusions:**

Standardized interruption protocols for DOAC therapy result in low or undetectable plasma levels in most patients undergoing high-risk elective orthopedic surgery. Routine preoperative DOAC level testing appears unnecessary in the general population but may be valuable in subgroups at high risk of bleeding.

## Introduction

Direct oral anticoagulants (DOACs), such as apixaban, rivaroxaban, edoxaban, and dabigatran, are commonly prescribed to reduce the risk of stroke in patients with atrial fibrillation and for the treatment and/or prevention of venous thromboembolism (VTE). Approximately 25% of patients on anticoagulant therapy require surgery within a 2-year period^[^1^]^. These patients typically need to pause their anticoagulant treatment before the procedure due to the increased risk of bleeding during or shortly after surgery.

Currently, it is not standard clinical practice to measure plasma levels of DOACs in patients undergoing elective surgery. Instead, a standardized interruption protocol is followed, based on the pharmacokinetics of each drug. A drug is generally considered eliminated from the body after five half-lives. However, the half-life of DOACs varies based on individual patient factors, such as renal function, age, drug interactions, and weight^[^2^]^. Since these drugs are cleared through the kidneys, impaired renal function, which is particularly common in elderly patients, can significantly impact drug elimination, although the effect of renal impairment on apixaban clearance is limited. Nevertheless, treatment should ideally be tailored to the individual patient^[^3^]^.

HIGHLIGHTS

- **Key findings**
  - Standard direct oral anticoagulants (DOAC) interruption resulted in low or undetectable preoperative plasma levels.
  - 38% of patients had undetectable DOAC levels before surgery.
  - 16% experienced non-major bleeding, mostly without detectable DOAC levels.
  - No major bleeding, thromboembolic events, or deaths occurred.
  - Routine DOAC level testing appears unnecessary, except in high-risk patients.
- **Background**
  - DOAC therapy is commonly interrupted before surgery to reduce bleeding risk. Current practice relies on standardized protocols rather than measuring drug levels, but real-world data on residual anticoagulant levels are limited.
- **Results and clinical relevance**
  - This study shows that standardized interruption protocols are effective and safe, with low residual drug levels and no thromboembolic events. Bleeding complications were not clearly linked to DOAC levels, suggesting other contributing factors. Routine testing is not required for most patients but may be useful in selected high-risk groups.

In surgical procedures with a low risk of bleeding, some residual anticoagulant activity may be acceptable. However, in a high-risk procedure, even minimal anticoagulant effects can be problematic. A previous study on dabigatran found that 85% of patients had no residual anticoagulant activity when the drug was interrupted 2–5 days before surgery, depending on renal function^[^4^]^. A subanalysis from the randomized evaluation of long-term anticoagulation therapy study demonstrated a strong correlation between dabigatran plasma levels and the incidence of thromboembolic events and major bleeding^[^5^]^. Shaw *et al*, in their meta-analysis, reported a 0.41% risk of thromboembolic events and a 1.81% risk of major bleeding within the first 30 days post-surgery among patients on anticoagulants^[^6^]^.

In theory, if the standardized interruption protocol is followed, plasma levels should be low at the time of surgery, thus minimizing the risk of perioperative bleeding. This study aims to contribute further insights into whether therapy interruption protocols should be individualized to better balance the risks of bleeding and thromboembolic events during and after elective surgery. This study has been reported in accordance with the STROCSS 2025 guidelines^[^7^]^.

## Methods

### Study design

This prospective observational study was conducted at Danderyd Hospital, Stockholm, Sweden, a university hospital with a catchment area of around 700 000 inhabitants. The study aimed to evaluate preoperative plasma levels of DOACs in patients undergoing elective orthopedic surgery associated with a high bleeding risk. The study period was from 1 May 2019 to 30 June 2022.

### Participants

Patients were eligible for inclusion if they were being treated with either a direct thrombin inhibitor (dabigatran) or a direct factor Xa inhibitor (apixaban or rivaroxaban) for atrial fibrillation or VTE and were scheduled for elective orthopedic surgery at Danderyd Hospital.

#### Inclusion criteria

- Age ≥ 18 years.
- Ongoing treatment with a DOAC (dabigatran, apixaban, or rivaroxaban) for atrial fibrillation or VTE.
- The patient is scheduled for elective orthopedic surgery associated with a high bleeding risk.
- Ability to provide written and oral informed consent.

#### Exclusion criteria

- Age less than 18 years.
- Terminal illness or an anticipated life expectancy of less than three months.
- Inability to provide informed consent (due to cognitive, language, or other barriers).

### Preoperative management

Preoperative interruption of DOAC therapy was performed in accordance with national guidelines established by the Swedish Medical Products Agency (Table 1)^[^8^]^. In the majority of cases, the timing and duration of the interruption were determined solely by adherence to the standardized protocol, which is based on the pharmacokinetic properties of each drug.

**Table 1:** Standardized interruption protocol.

	Dabigatran (Pradaxa)	Dabigatran (Pradaxa)	Apixaban (Eliquis), Rivaroxaban (Xarelto), Edoxaban (Lixiana)	Apixaban (Eliquis), Rivaroxaban (Xarelto), Edoxaban (Lixiana)
GFR (ml/min)	Low bleeding risk	High bleeding risk	High bleeding risk	Low bleeding risk
> 80	24 h	48 h	24 h	48 h
	Skip 2 doses	Skip 4 doses	Skip 1 dose rivaroxaban/edoxaban	Skip 2 doses rivaroxaban/edoxaban
			Skip 2 doses apixaban	Skip 4 doses apixaban
50–80	36 h	72 h	Same as above	Same as above
	Skip 3 doses	Skip 6 doses		
30–50	48 h	96 h	Same as above	Same as above
	Skip 4 doses	Skip 8 doses		
15–30	Contraindicated	Contraindicated	48 h	72 h
	Consult treating physician or coagulation specialist	Consult treating physician or coagulation specialist	Skip 2 doses rivaroxaban/edoxaban	Skip 3 doses rivaroxaban/edoxaban
			Skip 4 doses apixaban	Skip 6 doses apixaban
<15	Contraindicated	Contraindicated	Not recommended	Not recommended
	Consult coagulation specialist	Consult coagulation specialist	Consult coagulation specialist	Consult coagulation specialist

However, in patients who were deemed at higher risk of either bleeding or thrombosis, the interruption strategy was individualized and discussed with a coagulation consultant. This applied to patients meeting any of the following criteria:

- Impaired renal function is defined as a glomerular filtration rate (GFR) < 30 mL/min.
- Concomitant treatment with antiplatelet agents (e.g., acetylsalicylic acid, clopidogrel, or ticagrelor).
- Documented VTE within 3 months of surgery.
- Myocardial infarction, stroke, or percutaneous coronary intervention within 12 months prior to surgery.

### Blood sampling and laboratory analysis

A blood test was taken on the morning of surgery. The sample was centrifuged at 2000 *g* for 10 minutes and subsequently frozen. Analysis was performed after the last patient was included in the study. Hence, the drug concentration was not known during surgery. The levels of DOACs in plasma were measured using liquid chromatography–tandem mass spectrometry (LC–MS/MS)^[^9,10^]^.

### Data collection

Patient-specific data were collected through a review of electronic medical records up to 30 days postoperatively. Recorded variables included:

- Demographic data (age, sex, weight).
- Renal function (eGFR).
- DOAC type and timing of the last dose.
- Comorbidities.
- Concomitant use of non-steroidal anti-inflammatory drugs (NSAIDs) or selective serotonin reuptake inhibitors (SSRIs).
- Type of surgery and anesthesia.
- Postoperative use of blood transfusions or hemostatic agents.
- Any administration of reversal agents.
- Postoperative anticoagulation with low-molecular-weight heparin (LMWH).

### Outcomes

#### Primary endpoint

- Plasma DOAC concentrations on the day of surgery in patients managed according to standard interruption guidelines.

#### Secondary endpoints

- The incidence of bleeding or thromboembolic events within 30 days postoperatively.
- All-cause mortality within 30 days.

Thromboembolic events were defined as ischemic stroke, transient ischemic attack, myocardial infarction, systemic embolism, pulmonary embolism, or deep vein thrombosis. Bleeding events were classified according to the International Society on Thrombosis and Hemostasis criteria for major and clinically relevant non-major bleeding^[^11^]^.

### Anticoagulation strategy

Heparin bridging was not recommended, as previous studies have demonstrated an increased risk of bleeding without a corresponding reduction in thromboembolic events^[^12^]^. Postoperative thromboprophylaxis with LMWH was administered to most patients until DOAC therapy could be safely resumed, in accordance with institutional guidelines.

### Statistical analysis

Descriptive statistics were used to summarize baseline characteristics, surgical variables, and DOAC plasma concentrations. Continuous variables are presented as means and standard deviations for normally distributed data, and as medians with ranges for skewed data. Categorical variables are expressed as frequencies and percentages.

Plasma concentrations of DOACs were analyzed as continuous variables, and values below the lower limit of quantification (2.0 ng/mL) were treated as censored in descriptive summaries. Subgroup analyses were performed to explore potential associations between elevated plasma concentrations (> 30 ng/mL) and clinical factors such as renal function, age, and body weight. However, no inferential statistical testing (e.g., *t*-tests or regression modeling) was conducted due to the limited sample size, which would render the study underpowered for formal hypothesis testing.

All analyses were conducted using standard statistical software (e.g., IBM SPSS Statistics, version 30, or R, version 4.4.3), and graphical presentations were created using GraphPad (version 10.3.0).

## Results

### Study population

A total of 53 patients were included. There were notable interruptions to patient recruitment due to the COVID-19 pandemic and contractual problems with the testing laboratory, both of which delayed recruitment during certain periods (Fig. 1). The majority of patients were treated with apixaban; four patients received rivaroxaban, and three received dabigatran. The primary indication for anticoagulation was atrial fibrillation.

**Figure 1.**
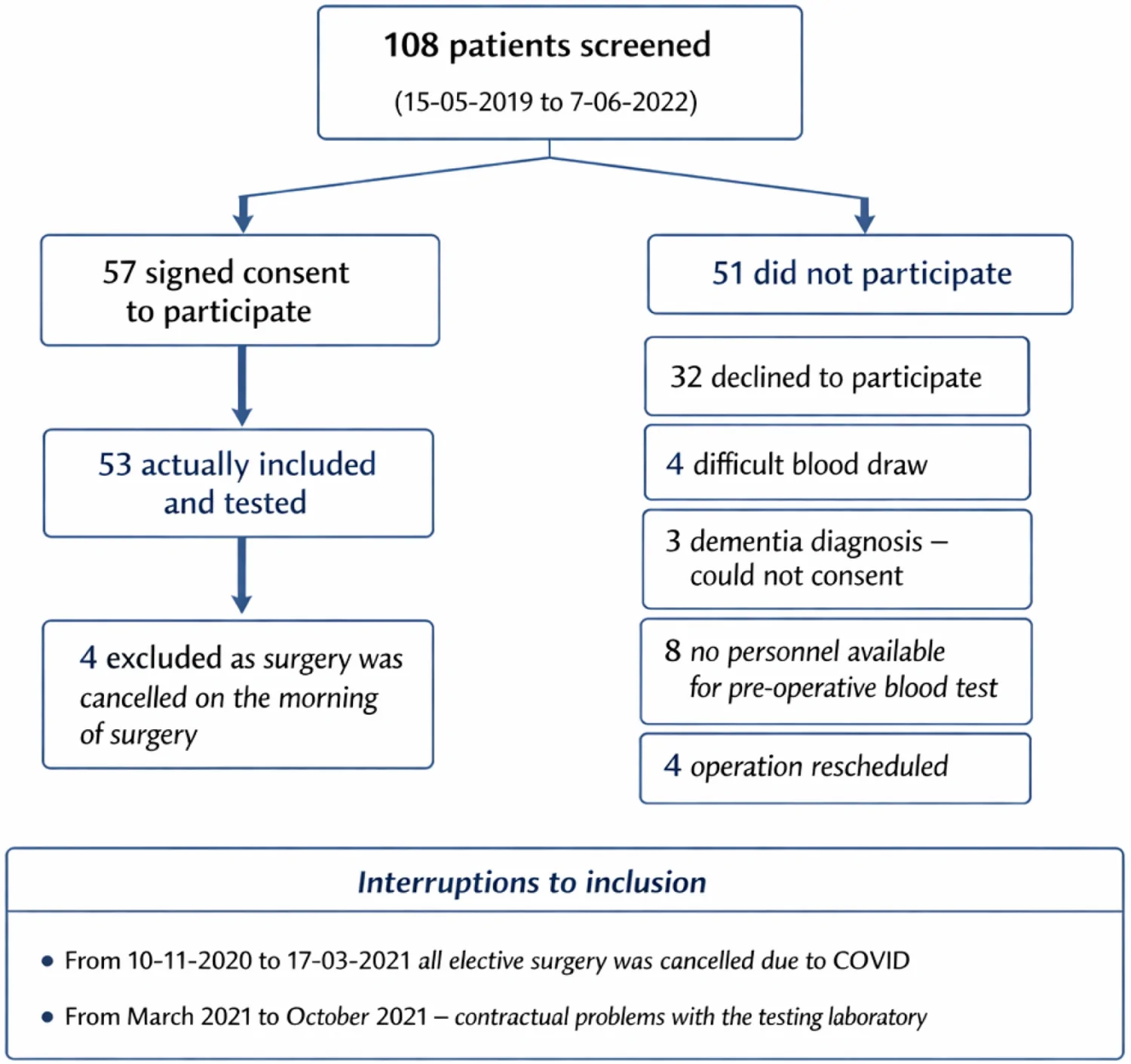
Patient screening and inclusion during the study period.

The mean age of the study population was 75.5 years (range: 59–89), with a slight predominance of male participants (54%). The median BMI was 29.1 kg/m^2^ (Table 2).

**Table 2:** Description of study population.

Age, mean (years)	75.5	Range 59–89
Male (*n*)	29	54%
Weight (kg)	87.1	Range 55–130
BMI (mean)	29.1	Range 20.3–40.4
eGFR (mean)	64,0	Range 31–90
>90	2	
60–89	28	
45–59	14	
30–44	7	
15–29	2	
Previous VTE (*n*)	3	10%
Medical history of cancer (*n*)	10	34%
CHADSVASc-score (mean)	3,8	Range 1–7
ASA (*n*)		
2	13	32%
3	41	68%
Type of DOAC (*n*)		
Apixaban	47	87%
Rivaroxaban	4	7%
Dabigatran	3	6%

### Surgical characteristics

The most common surgical procedures were hip replacement (33%) and total knee replacement (22%), followed by partial knee replacement (17%) and hip revision (18%). Most patients received spinal anesthesia (66%), with spinal plus epidural anesthesia used in 20% of cases (Table 3).

**Table 3:** Description of type of surgery and anesthesia.

Type of surgery		
Hip replacement surgery	18	33%
Total knee replacement surgery	12	22%
Partial knee replacement surgery	9	17%
Shoulder replacement surgery	5	9%
Hip revision surgery	10	18%
Type of anesthesia		
Spinal anesthesia	36	66%
Spinal + epidural anesthesia	11	20%
General anesthesia	2	4%
General + brachial plexus anesthesia	5	9%
Bleeding during surgery		
Mean, mL	260	Range 0–1100
Median, mL	200	Range 0–1100

### DOAC interruption timing

The duration of preoperative DOAC interruption ranged from 46 to 104 hours, with a mean of 78 hours and a median of 82 hours (Fig. 2). Time to resumption of therapy postoperatively ranged from 34 to almost 600 hours (mean: 77 h; median: 61 h) (Fig. 3). Prophylactic LMWH, at a standard dose 4500 IE of tinzaparin, was administered postoperatively in 90% of patients. Additionally, 94% of patients received intraoperative tranexamic acid, and in 14% (*n* = 4) this was continued postoperatively due to bleeding complications.

**Figure 2.**
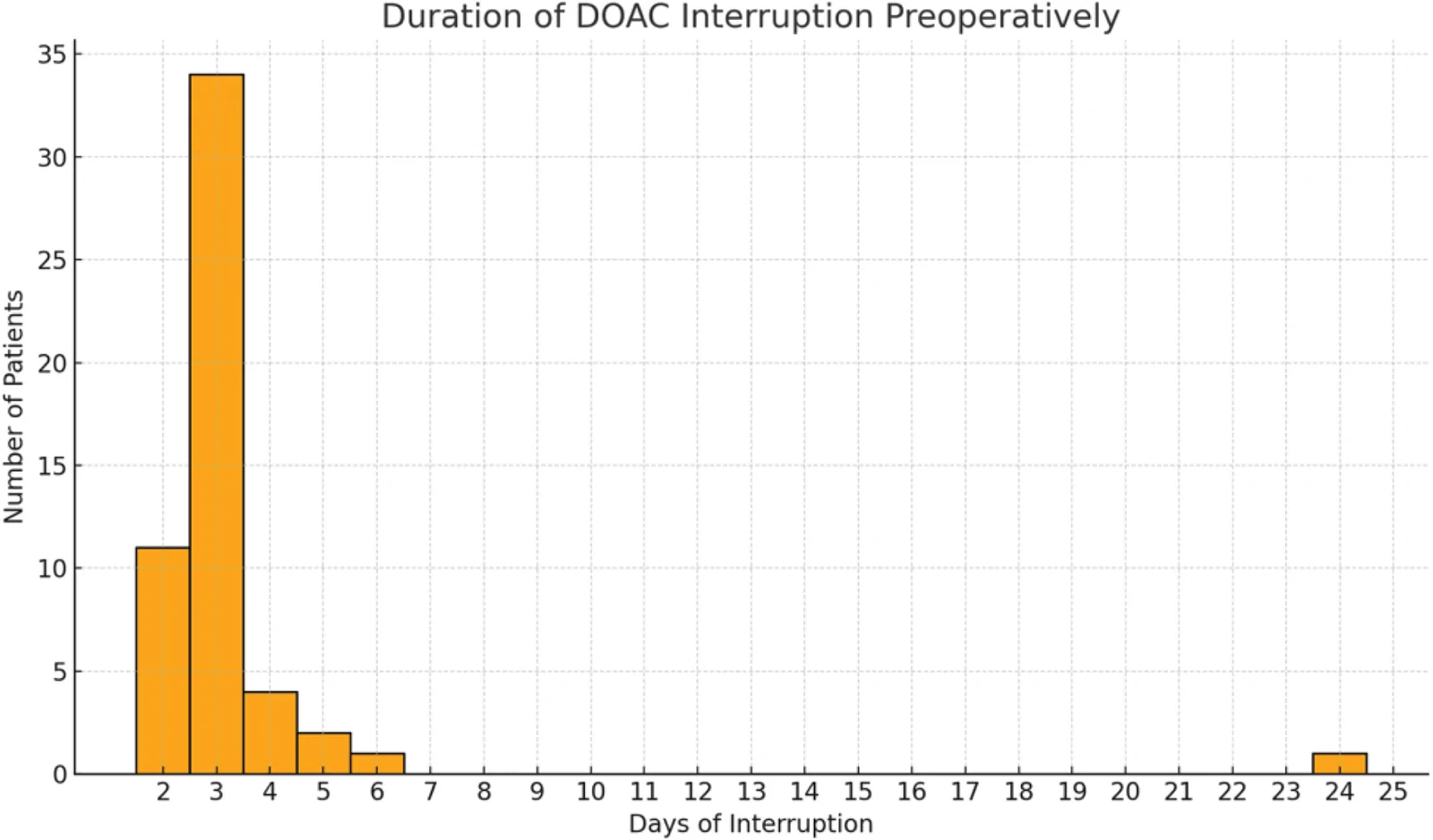
Duration of preoperative interruption of treatment.

**Figure 3.**
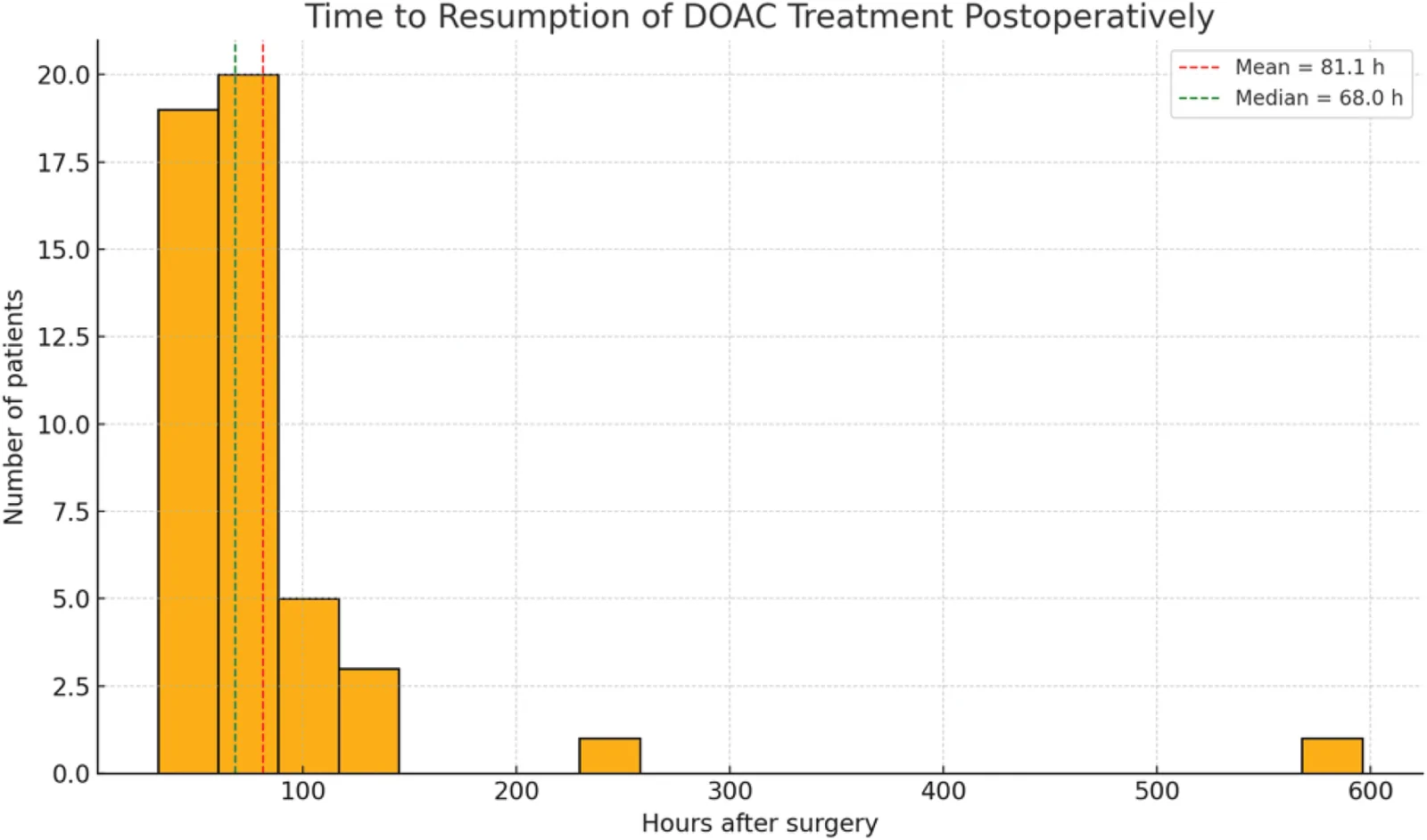
Time to DOAC resumption.

### DOAC plasma concentrations

Overall, DOAC plasma concentrations measured on the day of surgery were low. The mean concentration was 6.7 ng/mL, and the median was 3.7 ng/mL. In 38% of patients, the concentration was below the lower limit of quantification (<2.0 ng/mL) (Fig. 4). One patient with a weight of 130 kg had a pre-operative drug concentration of 4.9 ng/mL and resumed DOAC (apixaban) treatment within 48 hours.

**Figure 4.**
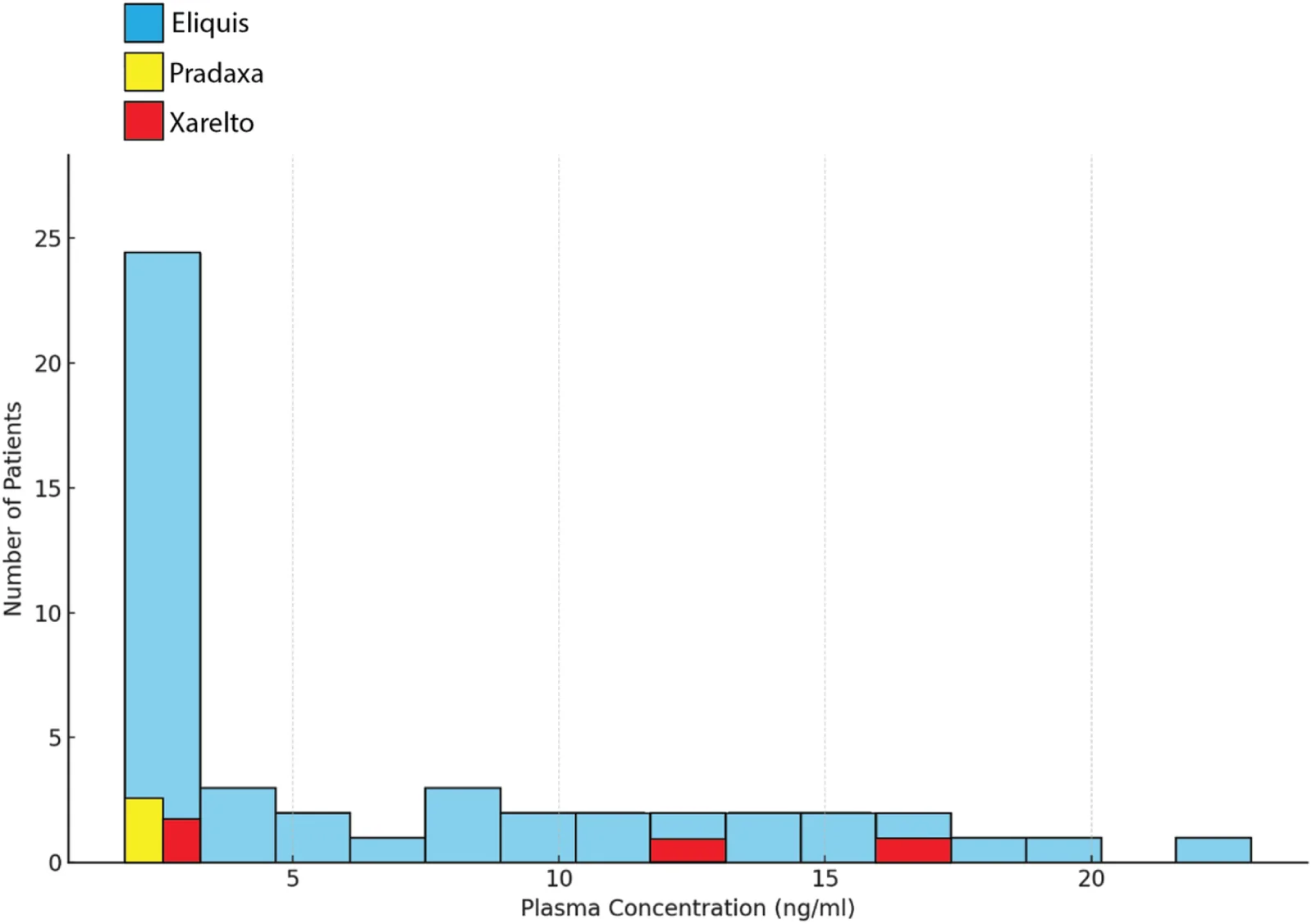
DOAC plasma concentrations.

### Bleeding and thromboembolic events

Clinically relevant non-major bleeding was observed in five patients (16%): Two patients developed postoperative hematuria. Three patients had bleeding at the surgical site. Notably, four of the five patients with bleeding had undetectable DOAC concentrations, while the fifth patient (on apixaban) had the highest observed concentration (23 ng/mL). This individual was 89 years old with impaired renal function (xx) and low body weight (yy) – factors known to increase the risk of DOAC accumulation. No major bleeding or thromboembolic events were reported. The 30-day postoperative mortality rate was 0%.

## Discussion

In this prospective observational study with a small sample size, we evaluated preoperative DOAC plasma concentrations in patients undergoing elective orthopedic surgery associated with high bleeding risk. Based on our findings, we suggest that the current standardized interruption protocol is largely effective, resulting in low or undetectable plasma drug levels in most patients^[^13^]^. These results align with existing recommendations and real-world data, supporting the clinical safety of standardized perioperative DOAC management.

The mean preoperative DOAC concentration in our cohort was 6.7 ng/mL, with a median value of 3.7 ng/mL, and in 38% of patients, the concentration was below the quantification threshold of 2.0 ng/mL. These low levels suggest effective elimination of the drug in the majority of patients prior to surgery. Importantly, only one patient, a 70 year-old woman on apixaban with normal renal function, had a concentration exceeding 20 ng/mL, nearing the upper safety threshold for surgery^[^14^]^. This reinforces the proposed cut-off level and highlights the need for vigilance in selected high-risk patients. This seems like a reasonable level, considering that the only patient in this study with a high plasma concentration, i.e., above this threshold, also had a bleeding complication after surgery.

Our study had a 16% rate of bleeding, which is higher than in most other studies. Four of the five patients with clinically relevant non-major bleeding had undetectable preoperative DOAC levels. Most patients with bleeding complications had hypertension, and one patient with elevated apixaban concentration also had impaired renal function and low body weight, both recognized risk factors for DOAC accumulation. Additional factors such as hypertension, concomitant medications, surgical complexity or duration, or perioperative tranexamic acid use may have contributed to bleeding risk, but our sample size was too small to draw any conclusions regarding these factors. We lacked information regarding NSAIDs or SSRIs, which are known to increase bleeding risk even in the absence of anticoagulants.

Despite the longer-than-recommended mean interruption period of 78 hours, compared to 48 hours in standard protocols for patients with normal renal function, no thromboembolic events were observed, and the 30-day postoperative mortality was 0%. While the absence of thrombotic complications is reassuring, the study’s limited size precludes definitive conclusions regarding the safety of prolonged interruption. Nonetheless, the longer interruption times may reflect clinician caution in the face of bleeding concerns, particularly in the elderly and those with comorbidities. The predominance of apixaban as the DOAC of choice is consistent with local guidelines and current literature supporting its favorable bleeding profile^[^15,16^]^. The relatively low number of patients on rivaroxaban and dabigatran limits our ability to compare drug-specific outcomes; however, similar pharmacokinetic principles apply across all DOACs, reinforcing the generalizability of our findings. One patient experienced a prolonged interruption period in excess of 500 hours due to prescription error; such cases are not uncommon, and prolonged interruption can also be due to post operative or other concerns^[^17^]^. A minority of patients underwent individualized interruption strategies based on clinician assessment. As these deviations may have influenced residual DOAC concentrations and bleeding outcomes, they represent a potential source of confounding and may limit interpretability of the findings.

Tripodi *et al* conclude in their review article^[^18^]^ that, while routine testing of DOAC levels is not necessary for all patients, it may be useful in selected cases, particularly in elderly patients or those with impaired renal function. The one patient in our study with elevated DOAC levels who also experienced a bleeding complication fits this profile, which further supports a risk-stratified approach. The low prevalence of high plasma concentrations in this study indicates that it is not necessary to regularly measure DOAC plasma concentrations prior to surgery. Our findings are in line with those from the perioperative anticoagulation use for surgery evaluation (PAUSE) study^[^19^]^, a large, prospective, multicenter trial that demonstrated the safety and efficacy of a standardized perioperative DOAC management strategy without the need for bridging anticoagulation or routine drug level testing. As in our cohort, the PAUSE study reported low residual DOAC levels and a low incidence of major bleeding and thromboembolic events. A recent review by Douketis and Spyropoulos also supports the safety of a standardized perioperative DOAC management strategy that omits bridging anticoagulation and routine drug level testing for most elective procedures^[^20^]^.

Routine measurement of DOAC concentrations may not be warranted in the general population; however, targeted testing may be prudent in high-risk subgroups. These may include elderly patients, those with significantly reduced renal function, low body weight, or those receiving concomitant antiplatelet therapy^[^21^]^. It should also be noted whether antiplatelet agents were paused preoperatively, as this may significantly influence bleeding risk; in our clinical practice, acetylsalicylic acid is typically continued, while clopidogrel is withheld for at least 5 days prior to surgery.

Additionally, the observation that most bleeding complications occurred in patients with undetectable DOAC levels underlines the importance of a comprehensive preoperative assessment that goes beyond drug elimination alone. Surgical complexity, tissue trauma, patient frailty, and polypharmacy must all be considered when estimating bleeding risk. In addition, all but one of the patients who experienced bleeding complications had a history of hypertension. Hypertension is associated with increased vascular stiffness, which may predispose patients to a higher risk of bleeding^[^22^]^.

Another notable finding is the high prevalence of tranexamic acid use as part of routine perioperative bleeding prophylaxis. While its efficacy in reducing blood loss is well established, future studies should assess how its use interacts with DOAC management strategies and whether it confers additional protection in patients with residual anticoagulant activity. It is worth noting that the interval between the last DOAC dose and surgery varies considerably in clinical practice. This variability may stem from ambiguities in how the recommendation to “discontinue DOAC 2 days before surgery” is interpreted, specifically, whether the last dose should be taken on the morning of preoperative day 2 or omitted entirely. A clearer instruction would be to specify the number of doses to withhold prior to surgery, depending on whether the patient is on a once-daily or twice-daily dosing regimen. Furthermore, the interruption of anticoagulation increases the potential for thromboembolic complications. Tripodi *et al* have suggested routine preoperative testing of DOAC concentrations to identify patients with high residual drug levels^[^23^]^. However, limited access to such testing and questions regarding its clinical value may hinder its widespread implementation^[^24^]^.

## Limitations

The main limitation of this study is its small sample size, which limits statistical power and generalizability. As an interim analysis, the findings are exploratory and hypothesis-generating rather than definitive. Enrollment was also temporarily paused during the COVID-19 pandemic due to the suspension of all elective surgeries, which further impacted recruitment and sample size. The observational design also introduces the possibility of confounding factors, and the lack of a control group limits causal interpretation. Moreover, the study was not designed to assess long-term outcomes or compare DOACs head-to-head.

Another limitation is that plasma DOAC concentrations were measured only once, on the day of surgery. Serial measurements before and after the procedure might have provided a more comprehensive pharmacokinetic profile and could have identified patterns of delayed clearance or unexpected accumulation. However, because most patients had low or undetectable levels on the day of surgery, serial testing after surgery would not have been applicable.

Further research is needed to refine perioperative DOAC management strategies, particularly in high-risk surgical populations. Larger, multicenter trials with sufficient statistical power ought to evaluate the predictive value of preoperative DOAC concentrations for bleeding and thrombotic events. These studies should also explore whether integrating drug-level testing into clinical protocols improves outcomes, especially when used selectively.

## Conclusion

Standardized interruption protocols for DOAC therapy result in low or undetectable plasma concentrations in most patients undergoing elective orthopedic surgery, with no observed thromboembolic events and only a few minor bleeding complications. Routine preoperative measurement of DOAC levels may not be necessary in all patients but could be considered in high-risk subgroups.

## Data Availability

The data are available from the corresponding author on reasonable request.
